# Heterotic gains, transgressive segregation and fitness cost of sweetpotato weevil resistance expression in a partial diallel cross of sweetpotato

**DOI:** 10.1007/s10681-023-03225-x

**Published:** 2023-09-27

**Authors:** Immaculate Mugisa, Jeninah Karungi, Paul Musana, Roy Odama, Milton O. Anyanga, Richard Edema, Paul Gibson, Reuben T. Ssali, Hugo Campos, Bonny M. Oloka, G. Craig Yencho, Benard Yada

**Affiliations:** 1 National Crops Resources Research Institute (NaCRRI), NARO, Kampala, Uganda; 2 Department of Agricultural production, Makerere University, Kampala, Uganda; 3 International Potato Center, Kampala, Uganda; 4 International Potato Center, Lima, Peru; 5 Department of Horticultural Science, North Carolina State University, Raleigh, NC USA; 6 National Crops Resources Research Institute (NaCRRI), P.O. Box 7084, Namulonge, Kampala, Uganda

**Keywords:** *Cylas* spp., Root yield, Dry matter, Clone, Cross, Sweetpotato

## Abstract

Heterosis-exploiting breeding schemes are currently under consideration as a means of accelerating genetic gains in sweetpotato (*Ipomoea batatas*) breeding. This study was aimed at establishing heterotic gains, fitness costs and transgressive segregation associated with sweetpotato weevil (SPW) resistance in a partial diallel cross of sweetpotato. A total of 1896 clones were tested at two sites, for two seasons each in Uganda. Data on weevil severity (WED), weevil incidence (WI), storage root yield (SRY) and dry matter content (DM) were obtained. Best linear unbiased predictors (BLUPs) for each clone across environments were used to estimate heterotic gains and for regression analyses to establish relationships between key traits. In general, low mid-parent heterotic gains were detected with the highest favorable levels recorded for SRY (14.7%) and WED (− 7.9%). About 25% of the crosses exhibited desirable and significant mid-parent heterosis for weevil resistance. Over 16% of the clones displayed superior transgressive segregation, with the highest percentages recorded for SRY (21%) and WED (18%). A yield penalty of 10% was observed to be associated with SPW resistance whereas no decline in DM was detected in relation to the same. Chances of improving sweetpotato through exploiting heterosis in controlled crosses using parents of mostly similar background are somewhat minimal, as revealed by the low heterotic gains. The yield penalty detected due to SPW resistance suggests that a trade-off may be necessary between maximizing yields and developing weevil-resistant cultivars if the current needs for this crop are to be met in weevil-prone areas.

## Introduction

Sweetpotato [*Ipomoea batatas* (L.) Lam], is an important food, nutrition, and income security crop in sub-Saharan Africa (SSA) (Anyanga et al. 2017; FAOSTAT 2020; Hotz et al. 2012). It is hailed as one of the crops with a high potential to contribute to combating hunger and malnutrition amidst increasing populations and climate stress in the region (Mwanga et al. 2017; Girard et al. 2021). The benefits accrue from its wide adaptability, natural hardiness, resilience to various stresses, and the fact that orange fleshed types are an affordable source of vitamin A (Hotz et al. 2012; Low et al. 2007; Mukasa 2004; Zhang et al. 2009). The productivity of this crop in SSA is however hampered by various abiotic and biotic constraints including, but not limited to the weevil complex, *Cylas puncticollis* and *Cylas brunneus* (Coleoptera: Brentidae). Sweetpotato weevils are known to cause losses ranging from 60 to 100% in marketable yield by feeding on the vines and storage roots (Anyanga et al. 2017; Fuglie 2007; Sorensen 2009). The severity of *Cylas* spp. varies with environment, with higher levels normally recorded in hotter areas that are more prone to dry spells (Smit et al. 2001; Sorensen 2009). In order to efficiently breed for resistance to sweetpotato weevils (SPWs), genotypes with higher levels of resistance combined with other quality attributes for instance high storage root yields and dry matter content, need to be identified and utilized in population improvement or hybridization.

Heterosis and transgressive segregation have played a key role in the success of plant breeding to date (Mackay et al. 2021). These two phenomena are responsible for yield increase in various crops globally over the past years, including maize, rice, and wheat (Li et al. 2008; Mackay et al. 2011; Troyer and Wellin 2009). Heterosis, also known as hybrid vigor, is a phenomenon whereby a filial 1 (F_1_) hybrid outperforms its parents (Bernardo 2014; Mackay et al. 2021). Transgressive segregation, on the other hand, refers to the occurrence of progeny with extreme phenotypes, that outperform their parents phenotypically in either a negative or positive way (Rieseberg et al. 1999). In their quest to improve specific traits, plant breeders normally seek to identify superior transgressive segregants and genotypes that outperform their best parents, either for more advanced testing and potential release or for deployment as parents in the next breeding cycle.

Heterosis is majorly attributed to non-additive effects like dominance, overdominance, and epistasis (Bingham 2015; Falconer and Mackay 1996; Jiang et al. 2017; Schnable and Springer 2013). It occurs when favorable dominant alleles from maternal and paternal lines accumulate in hybrids, subsequently leading to their better performance in comparison to the parental genotypes. In the case of overdominance, heterozygous F_1_ hybrids display superior performance over their homozygous parental lines due to favorable intra-locus allelic interactions. Epistasis, leads to heterosis when favorable underlying interactions occur between different loci, resulting in better hybrid performance (Jiang et al. 2017; Liu et al. 2020; Mackay et al. 2021; Schnable and Springer 2013). The phenomenon of transgressive segregation, on the other hand, is mostly ascribed to the complementary action of genes with additive effects. Levels of heterosis usually vary among and within different crop species, with cross-pollinated plants often displaying more heterosis compared to self-pollinated plants. Similarly, hybrids from genetically divergent parents have previously demonstrated greater degrees of heterosis compared to those that are from closely related parents (Liu et al. 2020). However, divergent genetic backgrounds may lead to reproductive isolation between two individuals (Ouyang and Zhang 2013).

Hybrid cultivars have historically played a vital role in enhancing productivity of both cross-pollinated and self-pollinated crops. Unlike cereal crops like maize and wheat in which big genetic gains have been achieved over the past couple of years using controlled crosses, (Laidig et al. 2014; Mackay et al. 2011; Troyer and Wellin 2009), heterosis in root crops such as sweetpotatoes, has not yet been fully exploited (Grüneberg et al. 2015; Nikiema 2016). There has been minimal focus on hybrid varietal development in breeding autopolyploids such as sweetpotato, since they are usually clonally propagated (Mackay et al. 2021), and sweetpotato breeding in the past was mainly done through polycross (Martin and Jones 2011; Grüneberg et al. 2015). However, preliminary investigations have shown that there is potential in achieving significant yield gains for sweetpotato roots and foliage through heterosis-exploiting breeding schemes and yield increments of over 20% have been reported when using controlled crosses and separate gene pools (Grüneberg et al. 2015). Controlled crosses enable the selection of parents on the basis of performance of their progeny, which eventually improves the efficiency of population improvement. Grüneberg et al. (2015), reported heterotic gains of up to 58% in storage root yield of sweetpotato clones that were progenies of 16 genotypes that had been crossed in a factorial design. Other researchers have equally attempted to estimate heterosis in sweetpotato on traits such as storage root yield, dry matter content, sweetpotato virus disease, flesh color, vine vigor, vine weight, and other quality traits (Aliou et al. 2020; Gurmu et al. 2018; Nikiema 2016). Nevertheless, studies on heterosis in sweetpotato are generally limited in comparison to other crops and there is limited information available on heterosis for sweetpotato weevil resistance in particular.

Researchers typically seek to enhance resistance to pests and diseases with minimal or no risk of fitness costs such as yield penalty to crops. However, pest and disease resistance is usually associated with fitness costs in host plants. Pedersen et al. (2005) defined fitness in relation to annual crops as, “The ability to produce economically harvestable yields of useful and commercially desirable plant products”. Many elements relating to a cultivar’s genotype and environment have an impact on fitness, including, but not limited to disease and pest resistance (Falconer 1989; Pedersen et al. 2005). Fitness cost of resistance can be considered as a negative correlation with another significant trait for instance yield, days to maturity or any other quality characteristic of a variety (Brown and Rant 2013). Such costs usually arise as a result of re-allocation of energy by the plant into activities related to self-defense, in the event of an attack by a bio-antagonist (Yang et al. 2012). Defense related activities may include the production of secondary metabolites (elicitors), modification of cell walls or induction of defense genes among others (Brown and Rant 2013). In rice, studies have shown that production of inducible defenses are given priority over plant growth through the jasmonate signaling pathway (Yang et al. 2012). The induction of jasmonic acid (JA) enhances protection against a variety of pests and pathogens by shifting resources away from growth and toward defense. (Yang et al. 2012). In breeding for resistance, if resistance to a given pest does actually have a negative impact on a crop’s potential yield, a balance must be struck between enhancing yields and reducing the expenses of pest control. However, the fitness costs associated with resistance to the African weevil species, *C. puncticollis* and *C. brunneus*, in relation to sweetpotato root yield and dry matter content are yet not clearly understood.

The present study was aimed at estimating heterotic gains and identifying desirable transgressive segregants in a diverse F_1_ sweetpotato population in an effort to distinguish the superior parental genotypes and F_1_ hybrids in terms of resistance to sweetpotato weevils, storage root yield and dry matter content. The study further sought to establish the extent to which SPW resistance influences the fitness of the crop with a focus on two major traits and performance indicators in sweetpotato, storage root yield and dry matter content.

## Materials and methods

### Study materials

The study made use of an F_1_ population known as the Mwanga Diversity Panel (MDP). This population was generated from an 8 × 8 paired cross between 16 parents, leading to 64 families and 1896 progenies (Table 1). The parental lines consisted of cultivars, breeding lines and landraces, most of which were sourced from within Uganda, with a small number of introductions (Zhou 2020; Mugisa et al. 2022).

**Table 1:** MDP parental lines

Code	Genotype	^a^Reaction to *Cylas* spp.	^b^Other attributes	Origin
*Male Parents*				
A1	‘Ejumula’	S	OFSP, creamy skin	Uganda
A2	NASPOT 1	S	CFSP, creamy skin	Uganda
A3	‘Dimbuka-Bukulula’	S	CFSP, creamy skin	Uganda
A4	NASPOT 5/58	S	OFSP, creamy skin	Uganda
A5	NASPOT 7	S	OFSP, creamy skin	Uganda
A6	SPK004 (Kakemega)	S	OFSP, purple-red skin	Kenya
A7	NASPOT 10 O	S	OFSP, purple-red skin	Uganda
A8	NK259L	MR	CFSP, purple-red skin	Uganda
*Female Parents*				
B1	‘Resisto’	S	OFSP, pinkish skin	USA
B2	‘Magabali’	MR	CFSP, creamy skin	Uganda
B3	NASPOT 5	MR	OFSP, creamy skin	Uganda
B4	‘Wagabolige’	MR	CFSP, creamy skin	Uganda
B5	‘Mugande’	S	OFSP, creamy skin	Uganda
B6	NASPOT 11	S	CFSP, purple-red skin	Uganda
B7	‘New Kawogo’	MR	CFSP, purple-red skin	Uganda
B8	‘Huarmeyano’	MR	CFSP, creamy skin	Peru

^a^*S* Susceptible, *MR* Moderately Resistant, ^b^*OFSP* Orange-fleshed sweetpotato, *CFSP* Cream-fleshed sweetpotato (Mugisa et al. 2022)

### Study sites and seasons

Field trials were established at two sites in Uganda, namely, Abi Zonal Agricultural Research and Development Institute (Abi-ZARDI) (3^o^4’37.2” N, 30^o^56’34.6” E; altitude, 1211 masl) and Ngetta Zonal Agricultural Development and Research Institute (Ngetta-ZARDI) (2^o^16’10.8’’N, 32^o^53’57.2’’ E; 1080 masl). Abi-ZARDI is located in North Western Uganda, receives about 1250 mm of annual total rainfall and has a mean temperature of 24 ^o^C. It is characterized by sandy clay loam soils (Kaizzi et al. 2012; Sserumaga et al. 2016). Ngetta ZARDI is located in Northern Uganda, receives 1361 mm of total rainfall annually with a mean temperature of 23.4 ^o^C. It is dominated by sandy loam types (Kaizzi et al. 2012; Wortmann and Eledu 1999). Trials in Abi-ZARDI were conducted in the first and second seasons of 2019 (2019 A and 2019B, respectively) whereas in Ngetta-ZARDI they were carried out in 2019B and in the first season of 2020 (2020 A). The two sites are located in different agro-ecological zones of the country and were purposively selected by virtue of their position in sweetpotato weevil hotspots.

### Study design and data collection

The trial was established as an augmented design with two checks: ‘New Kawogo’, as a resistant check and ‘Ejumula’ as the susceptible check. The two checks were randomly replicated in each of the 43 blocks at each field site. The 1896 progenies were evaluated together with their 16 parental genotypes and checks at each site. Planting was done with vines approximately 30 cm long on 3 m long ridged plots. A spacing of 30 cm was maintained between plants and 1 m between ridges. Harvesting was done at 5 months after planting instead of the 4 months usually required for maturity, to give ample time for the weevil population to build up.

At harvest, data was collected on vine weight and weight of both marketable and non-marketable storage roots (kg) per plot. Data was also taken on the total number of roots and number of infested roots per plot. Weevil incidence (WI) was determined by expressing the number of infested roots in each plot as a percentage of the total number of roots. Weevil severity (WED) was assessed by inspection of harvested storage roots in each plot followed by scoring using a scale of 1 to 9, where: 1 = no damage; 3 = minor; 5 = moderate; 7 = heavy; and 9 = severe damage, with numbers in between rep-resenting intermediate ratings (Grüneberg et al. 2019).

The percentage dry matter content (DM) of each genotype was obtained by slicing two randomly selected clean medium-sized (approximately 200–300 gm) fresh storage roots of that genotype into small chips. Thereafter, a sub-sample (100–200 gm) was obtained and weighed to determine the fresh weight. The sub-sample was then placed in a well-labeled paper bag, and dried at 70 ^o^C in an oven for 72 h until a constant mass was attained. The dry mass was finally weighed and the weight was expressed as a percentage of the fresh weight (Islam et al. 2002).

#### Data analysis

Data were analyzed in R statistical software, v. 4.0 (R Core Team 2020) using the *augmentedRCBD* function in the R package “*agricolae*” (v. 4.1.2). Progeny with missing data were excluded from the analysis since some were unable to survive in the field and others had very poor field establishment. Each combination of site and season was considered as a unique environment, giving a total of four environments. Additionally, best linear unbiased predictors (BLUPs) were extracted for each clone using the *ranef* function in the *lme4* package (Bates et al. 2015) and used to compute heterotic gains across all environments for all traits.

Mid-parent heterotic gains (MPH) for the various traits were calculated as follows:

$$MPH\left(\%\right)=\left[\frac{{F}_{1}-MP}{MP}\right]\times 100$$

where: F_1_ is the mean of the F_1_ hybrid and MP is the mean performance of the parental lines (Falconer and Mackay 1996; Grüneberg et al. 2015; Hochholdinger and Hoecker 2007); High-parent heterotic gains (HPH) were calculated as follows:

$$\varvec{H}\varvec{P}\varvec{H}\left(\varvec{\%}\right)=\left[\frac{{\varvec{F}}_{1}-\varvec{H}\varvec{P}}{\varvec{H}\varvec{P}}\right]\varvec{\times} 100$$

where: F_1_ is the mean of the F_1_ hybrid, and HP is the mean of the superior (best) parent lines (Falconer and Mackay 1996; Grüneberg et al. 2015; Hochholdinger and Hoecker 2007).

The difference between the predicted means of the F_1_ clones of a particular cross and the mid-parents and high-parents were tested using a t-test. The occurrence of transgressive segregation for weevil severity, weevil incidence, storage root yield and dry matter content was likewise assessed based on predicted means for the respective traits. Chi-square tests were performed to compare the number of clones expected to exceed the means of each parent by to the observed number that actually surpassed them (Rodríguez et al. 2005).

In order to establish fitness costs of SPW resistance on SRY and DM, BLUPs were used to perform separate simple linear regression analyses between each of these traits and WED using the package “*Psych*” in R statistics software. With unbalanced datasets, BLUPS are known to yield more accurate estimates of genotype performance (Piepho et al. 2008). Finally, the Summa rank-based summation index was employed to identify superior genotypes based on their mean performance in terms of weevil severity, incidence, storage root yield, and dry matter content (Mulamba and Mock 1978).

## Results

Trait variations and performance of clones and families.

**Fig. 1:**
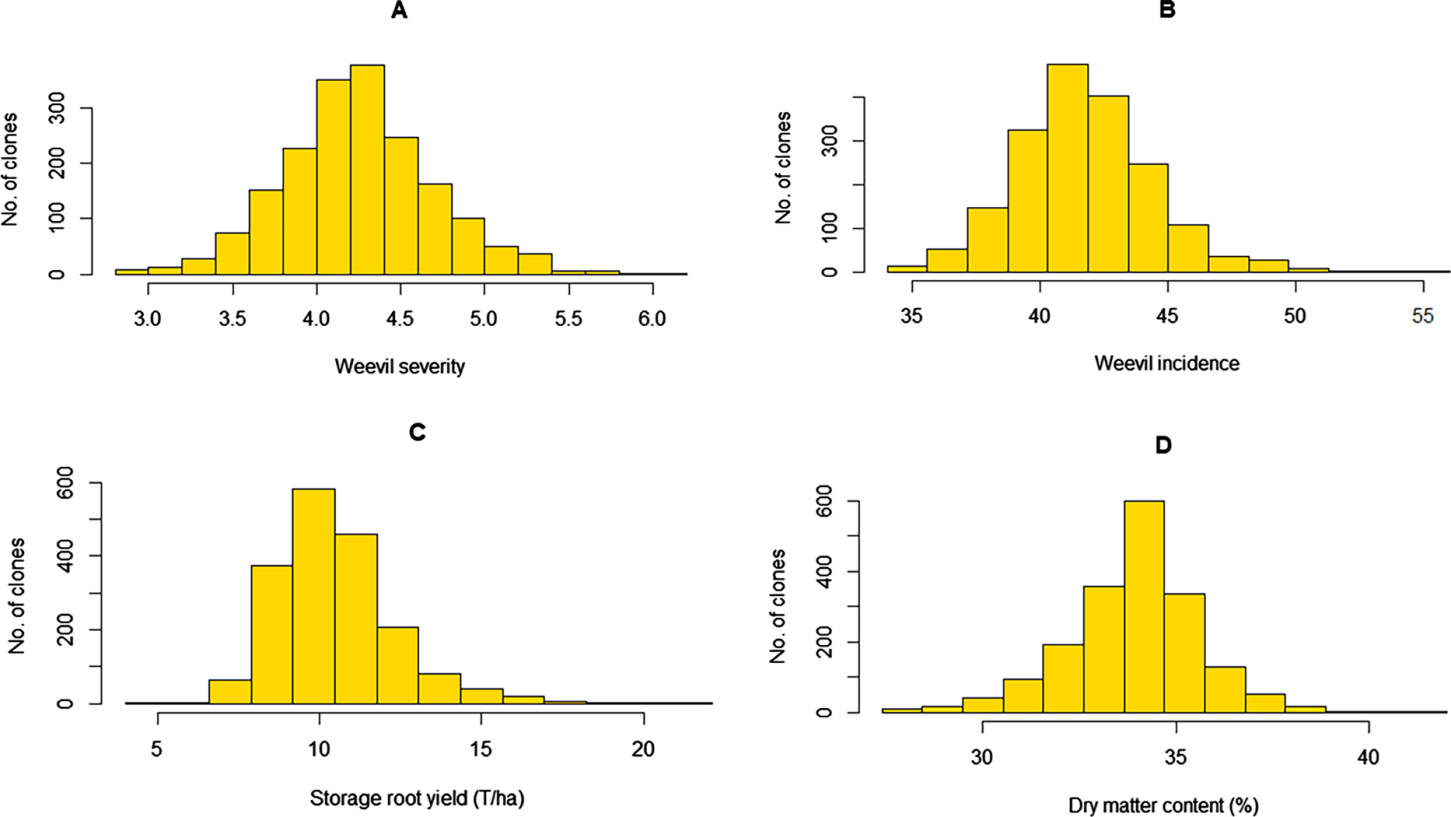
Distribution of mean **A** weevil severity; **B** weevil incidence; **C** storage root yield; and **D** dry matter content in the MDP population

Significant differences were recorded in genotypes, families and environments for all traits (Table 2). However, the genotype*environment and family*environment interactions did not have a significant effect on all traits. All traits showed a normal distribution (Fig. 1). Overall means obtained from the test population for WED, WI, SRY and DM were 4.2, 35.7%, 11.6 T/ha, and 33.8%, respectively.

**Table 2:** Mean squares for weevil severity, weevil incidence, storage root yield and dry matter content for 64 sweetpotato families evaluated in multi-environments

Source of variation	Mean Squares				
	df	WED	WI	SRY	DM
Genotype (G)	1514	5.30***	0.43**	0.13***	0.30***
Family (F)	63	8.10***	0.58***	0.26***	0.94***
Environment (E)	3	1076.2***	128.3***	72.25***	42.28**
Block (B)	42	2.30	0.24	0.12*	0.20
G × E	2418	3.00	0.21	0.07	0.15
F × E	189	3.40	0.20	0.08	0.15
B × E	126	8.30***	0.43*	0.30***	0.26*
Residual	88	3.30	0.25	0.08	0.18

*df* Degrees of freedom, *WED* Weevil severity, *WI* Weevil Incidence, *SRY* = Storage root yield (T/ha), *DM * Dry matter content (%), *Significant at *P* < 0.05, **Significant at *P* < 0.01; ***Significant at *P* < 0.001

**Fig. 2:**
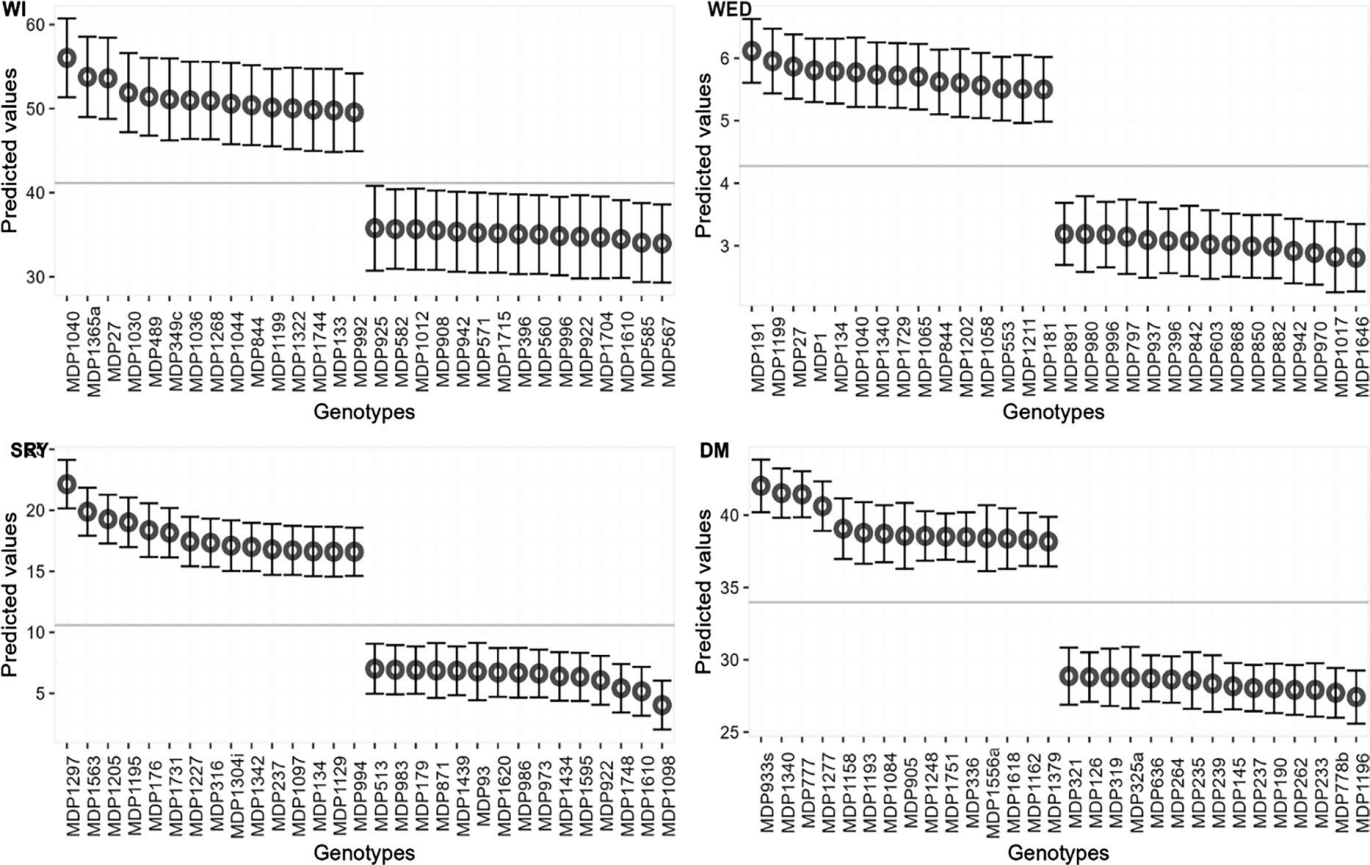
Best and worst performing clones based on BLUPs for weevil severity (WED), weevil incidence (WI), storage root yield (SRY) and dry matter content (DM)

The best and worst performing clones for the traits studied based on predicted means are presented in Fig. 2. Clones “MDP996” and “MDP942” were among the best performers for both weevil severity and incidence across environments whereas “MDP844”, “MDP1040”, and “MDP27” featured among the worst. Superior clones in terms of SRY and DM were “MDP1297” (22.1 T/Ha) and “MDP933s” (42%), respectively.

**Fig. 3:**
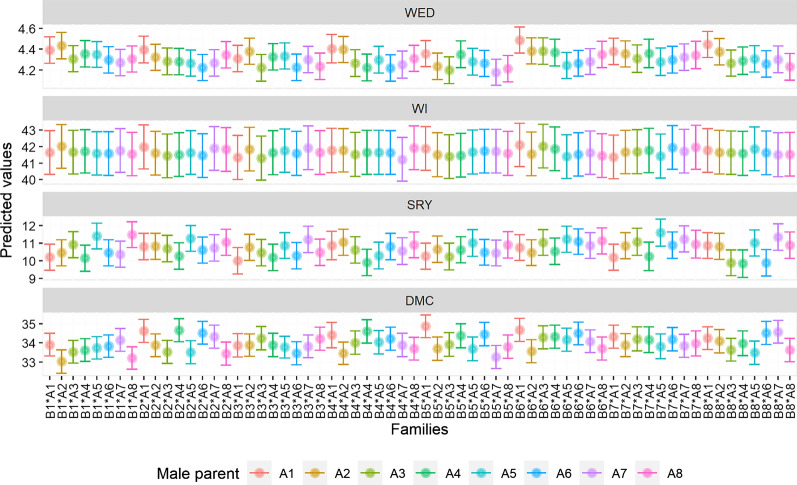
Trait means of the sixty-four families based on BLUPs across four environments

Considering the sixty-four families, ‘Mugande’ x NASPOT 10 O (B5*A7), ‘Mugande’ x ‘Dimbuka-bukulula’ (B5*A3) and ‘Mugande’ x NK259L (B5*A8), had the lowest predicted means for weevil damage (Fig. 3). In terms of weevil incidence, superior families included ‘Wagabolige’ x NASPOT 10 O (B4*A7), NASPOT 5 x ‘Dimbuka-bukulula’ (B3*A3), NASPOT 5 x ‘Ejumula’ (B3*A1), and ‘New Kawogo’ x ‘Ejumula’ (B7*A1). Families with the highest mean SRY and DM were ‘New Kawogo’ x NASPOT 7 (B7*A5) and ‘Mugande’ x ‘Ejumula’ (B5*A1), respectively.

### Mid-parent and High-parent heterotic gains.

Families that displayed the most favorable mid-parent and high-parent heterotic gains for weevil severity included ‘Mugande’ x NASPOT 10 O (− 7.92%, − 5.75%) and ‘Mugande’ x ‘Dimbuka-bukulula’ (− 7.53%, − 5.34%) respectively (Table 3). With respect to weevil incidence, NASPOT 5 x ‘Dimbuka-bukulula’ (− 5.73%, − 4.86%) and ‘Mugande’ x ‘Dimbuka-bukulula’ (− 5.65%, − 4.11%) had the most favorable MPH and HPH levels respectively. The highest MPH for SRY and DM were recorded in ‘New Kawogo’ x NASPOT 7 (14.75%), and NASPOT 11 x ‘Ejumula’ (3.68%) respectively. Families that displayed the highest HPH for SRY and DM included ‘New Kawogo’ x NASPOT 7 (9.12%), ‘Mugande’ x ‘Ejumula’ (3.52%). Generally, heterosis relative to both mid-parents and high-parents was greater among the moderately resistant x susceptible crosses than among the susceptible x susceptible crosses (Table 5). The cross NASPOT 11 x ‘Ejumula’, exhibited the least desirable heterotic gains for both weevil severity and incidence across all sites. Out of the 64 families tested, more than 25% exhibited desirable and significant MPH for weevil severity and incidence. An estimated 20% of the crosses were significantly better than the mid-parent in terms of SRY and DM (Table 3). Only one cross, NASPOT 11 x NASPOT 7 was superior, and displayed desirable heterosis for all the traits. In general, heterotic gains observed were highest or most favorable for storage root yield, followed by weevil severity, weevil incidence, and dry matter content (Table 3).

**Table 3:** Estimates of percentage mid-parent and high-parent heterotic gains for weevil severity, weevil incidence, root yield and dry matter content in the sixty-four families

	WED		WI		SRY		DM	
Family	MPH%	HPH%	MPH%	HPH%	MPH%	HPH%	MPH%	HPH%
Resisto x Ejumula	5.3**	6.9**	2.1	3.0*	-7.5***	-7.7*	-2.6	− 4.9
Resisto x NASPOT 1	6.2**	7.8***	1.8	3.5**	− 3.9	− 4.5	− 6.3**	− 8.4**
Resisto x Dimbuka-B	0.9	2.5*	− 3.5^***^	1.1	− 0.7	− 1.1	− 4.6*	− 6.8**
Resisto x NASPOT5/58	2.4*	4.0**	− 1.8	1.1	− 9.7**	− 10.0**	− 3.6	− 5.9
Resisto x NASPOT 7	3.1*	4.8**	− 2.0*	1.7	8.9***	7.4***	− 4.3*	− 6.5*
Resisto x SPK004	0.7	2.4*	− 1.2	2.1	− 5.8**	− 6.1*	− 2.9*	− 5.2*
Resisto x NASPOT 10 O	− 0.9	0.6	1.5	3.9*	− 5.3	− 6.1*	− 2.5	− 4.7*
Resisto x NK259L	0.3	2.0	0.1	2.2	5.4**	4.2	− 5.8	− 7.9*
Magabali x Ejumula	3.7*	4.1**	4.4^*^	5.5**	0.4	− 2.6	2.5*	2.9*
Magabali x NASPOT 1	0.2	0.6	1.3	1.6	2.1	− 0.6	− 0.5	− 1.1
Magabali x Dimbuka− B	− 2.5*	− 2.1*	− 3.9^***^	− 1.1	0.5	− 2.3	− 1.2	− 1.6
Magabali x NASPOT5/58	− 2.4*	− 2.1*	− 1.6	− 0.6	− 4.6*	− 8.1**	2.4*	2.0*
Magabali x NASPOT 7	− 2.2*	− 1.8*	− 1.3	0.4	10.5**	8.3**	− 1.6	− 2.1
Magabali x SPK004	− 4.3***	− 4.0**	− 0.9	0.5	− 1.8	− 4.7	2.4*	2.0
Magabali x NASPOT 10 O	− 3.3*	− 3.0*	2.9	3.4	0.9	− 1.5	1.3	0.9
Magabali x NK259L	− 0.2	0.1	1.8	2.0	3.7	1.4	− 1.9	− 2.4*
NASPOT 5 x Ejumula	0.3	1.1	0.7	5.7*	− 14.2*	− 18.9**	− 0.2	− 0.9
NASPOT 5 x NASPOT 1	2.9*	3.7*	1.0	5.3	− 6.2	− 11.7**	− 1.4	− 1.9*
NASPOT 5 x Dimbuka-B	− 4.5***	− 3.8*	− 5.7***	− 4.8**	− 10.9**	− 16.0*	− 0.2	− 0.8
NASPOT 5 x NASPOT5/58	− 0.1	0.5	− 2.4*	0.1	− 15.2**	− 19.4**	− 0.4	− 1.1
NASPOT 5 x NASPOT 7	0.8	1.5	− 2.0*	− 0.1	0.7	− 5.7*	− 1.7	− 2.2
NASPOT 5 x SPK004	− 3.9*	− 3.3*	− 1.6	0.6	− 13.1**	− 17.9**	− 1.2	− 1.9*
NASPOT 5 x NASPOT 10 O	− 1.7	− 1.1	1.8	5.3*	− 3.2	− 9.1*	− 0.9	− 1.6
NASPOT 5 x NK259L	− 3.8**	− 3.1*	0.2	3.8*	− 8.2	− 14.0**	− 0.8	− 1.3
Wagabolige x Ejumula	6.2***	6.8**	3.0	6.3**	− 1.1	− 3.3*	2.9*	3.1**
Wagabolige x NASPOT 1	2.7*	3.2*	0.7	3.1*	− 0.8	− 3.4*	− 1.5	− 1.98*
Wagabolige x Dimbuka-B	− 2.7*	− 2.3*	− 4.6***	− 3.9*	− 4.7*	− 7.0*	0.2	− 0.1
Wagabolige x NASPOT5/58	− 4.3***	− 3.9*	− 2.4*	− 1.2	− 12.6**	− 14.1**	2.8*	3.1**
Wagabolige x NASPOT 7	− 1.2	− 0.7	− 2.3*	− 2.1**	− 1.5	− 4.7*	0.2	− 0.2
Wagabolige x SPK004	− 4.5**	− 4.1**	− 1.3	− 0.7	− 4.9**	− 7.1**	1.6	1.9*
Wagabolige x NASPOT 10 O	− 3.6*	− 3.2*	− 0.6	0.9	− 5.0*	− 7.7*	0.5	0.8
Wagabolige x NK259L	− 0.6	− 0.2	1.3	3.2*	− 0.6	− 3.7	− 1.0	− 1.4
Mugande x Ejumula	0.6	3.1*	2.7*	8.6**	− 9.9*	− 13.4**	3.5**	3.5***
Mugande x NASPOT 1	− 4.5**	− 2.1*	− 0.3	4.5*	− 5.9*	− 10.0*	− 1.1	− 0.9
Mugande x Dimbuka-B	− 7.5***	− 5.3**	− 5.6**	− 4.1**	− 9.7*	− 13.5**	0.1	0.2
Mugande x NASPOT5/58	− 0.9	1.4	− 3.3**	− 0.0	− 8.5*	− 11.7*	1.8	1.9
Mugande x NASPOT 7	− 3.4*	− 1.1	− 2.6**	0.0	3.5**	− 1.6	− 1.1	− 1.3
Mugande x SPK004	− 4.1**	− 1.8*	− 1.3	1.6	− 8.7*	− 12.4**	2.3*	2.3*
Mugande x NASPOT 10 O	− 7.9***	− 5.7**	0.6	4.8*	− 6.6*	− 10.8**	− 1.2	− 1.1
Mugande x NK259L	− 6.5***	− 4.3*	− 0.2	4.2*	− 2.1*	− 6.7**	− 0.7	− 0.9
NASPOT 11 x Ejumula	9.8**	11.7***	6.0*	7.4**	0.8	− 5.3*	3.6**	2.6*
NASPOT 11 x NASPOT 1	4.9*	6.8*	2.3*	4.4*	− 0.2	− 5.9**	− 0.8	− 2.1
NASPOT 11 x Dimbuka− B	4.9**	6.9**	0.5	5.6*	2.8*	− 3.7*	1.5	0.2
NASPOT 11 x NASPOT5/58	4.1*	6.0**	1.5	4.7*	− 1.9*	− 8.4**	2.2*	1.1
NASPOT 11 x NASPOT 7	− 0.2	1.6	− 0.6	3.6*	10.3**	4.6*	0.7	− 0.4
NASPOT 11 x SPK004	0.5	2.5*	0.9	4.9**	2.8*	− 3.3*	3.0**	1.9*
NASPOT 11 x NASPOT 10 O	0.2	2.1*	3.2*	6.1***	2.9*	− 2.7*	1.5	0.4
NASPOT 11 x NK259L	2.8*	4.8*	1.9	4.5**	5.3***	− 0.3	− 0.3	− 1.4
New Kawogo x Ejumula	3.6*	4.8*	2.5*	3.6*	− 2.2*	− 7.9**	1.6	1.2
New Kawogo x NASPOT 1	2.4*	3.6*	2.1*	3.9**	3.9*	− 1.8	− 1.0	− 1.5
New Kawogo x Dimbuka-B	− 0.6	0.5**	− 2.4**	2.6*	5.0**	− 1.0	− 0.0	− 0.4
New Kawogo x NASPOT5/58	5.2*	6.4**	1.2	4.5**	− 8.6*	− 14.4**	1.1	0.7
New Kawogo x NASPOT 7	− 1.1	0.1	− 1.6	2.3*	14.7***	9.1**	− 1.2	− 1.7
New Kawogo x SPK004	− 0.3	0.8	1.5	5.3**	1.9	− 4.0	1.3	0.9
New Kawogo x NASPOT 10 O	− 0.1	1.1	2.6*	5.3*	6.2*	0.5	− 0.2	− 0.6
New Kawogo x NK259L	0.7	1.9*	3.0	5.4*	5.4**	0.0	− 0.8	− 1.3
Huarmeyano x Ejumula	7.4**	7.5***	3.2*	6.5**	− 2.2*	− 6.5**	1.8*	1.9*
Huarmeyano x NASPOT 1	2.8*	2.9*	1.0	3.5	− 2.3*	− 6.9**	− 0.0	− 0.3
Huarmeyano x Dimbuka-B	− 1.4	− 1.2	− 3.2**	− 2.5*	− 10.9*	− 15.0**	− 0.6	− 0.8
Huarmeyano x NASPOT5/58	− 0.4	− 0.3	− 1.7	− 0.8	− 13.5*	− 16.8**	0.8	0.7
Huarmeyano x NASPOT 7	0.6	0.7	− 0.6	− 0.3	4.5*	− 1.0	− 1.4	− 1.7
Huarmeyano x SPK004	− 1.4	− 1.2	− 0.5	0.1	− 12.4*	− 16.3**	2.6**	2.5**
Huarmeyano x NASPOT 10 O	− 0.2	− 0.0	1.2	2.87*	1.3	− 3.7*	2.3**	2.1*
Huarmeyano x NK259L	− 2.8*	− 2.7*	0.6	2.61*	− 0.8	− 5.9*	− 1.3	− 1.5

*
MPH* Mid-parent heterotic gains, *HPH* High-parent heterotic gains, *WED* Weevil severity, *WI* Weevil Incidence, *SRY* Storage root yield (T/ha), *DM * Dry matter content (%), *Dimbuka-B* Dimbuka- bukulula;,***Significant at *P* < 0.001, **P < 0.01; *P < 0.05

### Transgressive phenotypes

**Fig. 4:**
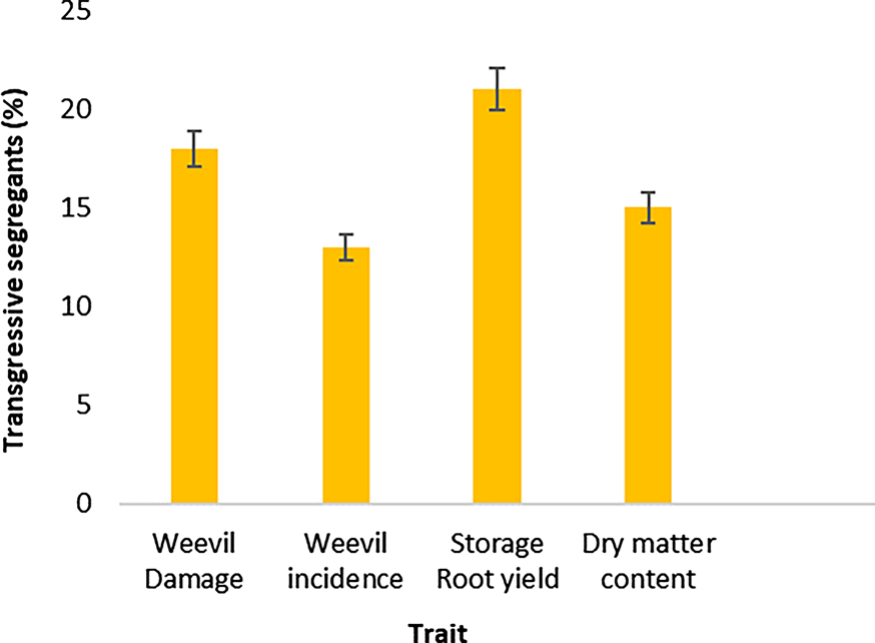
Percentage of superior transgressive segregants per trait

Clones that surpassed the parental limits in terms of better performance were observed in all families and traits. The highest percentage of superior transgressive segregants were recorded for storage root yield (21%) and WED (18%) (Fig. 4). Traits that displayed less superior transgressive segregation included dry matter content (15%) and weevil incidence (13%). Out of the 1896 clones tested, only 4.5% displayed superior transgressive segregation for all traits. Some families with the highest number of superior segregants for WED also had more superior segregants for weevil incidence. These included NASPOT 11 x NASPOT 7, ‘Wagabolige’ x NASPOT 10 O, and ‘Resisto’ x SPK004. Notably, crosses in which NASPOT 11 was the female parent had higher numbers of positive transgressive segregants for storage root yield and dry matter content. The cross that involved the two checks used in this study; ‘New Kawogo’ and ‘Ejumula’, was similarly among those that produced the highest numbers of desirable segregants for weevil incidence, and storage root yield. In general, the crosses that displayed the highest total number of superior transgressive segregants for all traits were NASPOT 11 x NASPOT 7, ‘Resisto’ x NASPOT 7, NASPOT 11 x SPK004 and ‘New Kawogo’ x ‘Ejumula’.

### Fitness cost associated with sweetpotato weevil resistance expression

**Fig. 5:**
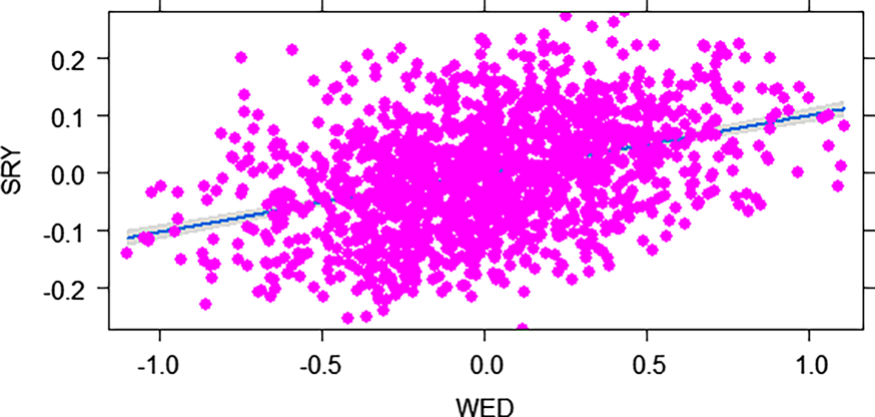
Relationship between storage root yield (SRY) and weevil severity (WED) in the MDP population

A fairly low positive significant association was observed between SRY and WED (r^2^ = 0.144; *p* value < 2.2e-16 ***; *y* = − 0.0012 + 0.1*x)* (Fig. 5). The r^2^ value obtained indicates that 14.4% of the variability observed in SRY is attributed to WED. We found that SRY tends to decrease with decreasing values of WED, implying that clones that express resistance (having less weevil damage), are generally reporting less root yield than those that are susceptible. The regression coefficient (0.1) reveals that for every unit decrease in WED, there is a 10% corresponding decrease in SRY. On the other hand, no relationship was detected between DM and WED (r^2^ = 0.001; *p* value = 0.104; *y* = 0.001 + -Fitness cost associated with sweetpotato weevil resistance expression.0.01*x*).

### Best performing genotypes based on rank summation index

Breeders are usually interested in improving target traits without negatively influencing the performance of other farmer preferred non-target traits. High storage root yield and dry matter content are among the key farmer preferred traits in sweetpotato and are usually evaluated alongside weevil resistance (Grüneberg et al. 2019). The summa rank-based selection index, proposed by Mulamba and Mock (1978), was used to identify the superior clones based on the actual means of the four traits. The ranking was done while putting into consideration the minimum preferred trait values in the breeding program at NaCRRI: WED < 2; SRY > 17 tons/ha and DM > 28% (Odama 2021). Ranking of genotypes revealed the superior genotypes for all traits combined (Table 4). Overall best performers were MDP449, MDP1514 and MDP920.

**Table 4:** Best performing clones (20) based on the summa rank-based selection index

No.	Genotype	WED	Rank	WI	Rank	RY	Rank	DM	Rank	RSI
1	MDP449	1	1	0	1	19	5	44	1	8
2	MDP1514	1	1	0	1	14	7	38	3	12
3	MDP920	1	1	0	1	16	6	35	6	14
4	MDP797	1	1	0	1	13	10	37	4	16
5	MDP43	1	1	0	1	20	4	33	10	16
6	MDP904	1	1	0	1	26	1	32	14	17
7	MDP1386	1	1	0	1	12	11	37	5	18
8	MDP1336	1	1	0	1	14	8	34	9	19
9	MDP1644	1	1	0	1	22	3	31	15	20
10	MDP1124	1	1	0	1	12	12	35	7	21
11	MDP1706	1	1	0	1	22	2	28	19	23
12	MDP1066	1	1	0	1	7	20	41	2	24
13	MDP1334	1	1	0	1	11	13	33	12	27
14	MDP1293a	1	1	0	1	13	9	30	18	29
15	MDP1656c	2	3	0	1	9	18	35	8	30
16	MDP106b	1	1	0	1	9	17	33	11	30
17	MDP213	1	1	0	1	10	15	32	13	30
18	MDP1704	2	3	0	1	10	16	31	16	36
19	MDP686	1	1	0	1	11	14	26	20	36
20	MDP793	1	1	0	1	8	19	31	17	38

*WED* Weevil severity, *WI* Weevil Incidence, *SRY* Storage root yield (T/ha), *DM * Dry matter content (%), *RSI* Rank summation index

## Discussion

The clones that were tested exhibited varying levels of resistance and susceptibility to sweetpotato weevils. The significant differences detected between these clones in weevil severity and weevil incidence suggest that these traits could be genetically enhanced for weevil resistance through selection of better performers. The variation in their performance could be due to different factors including differences in morphological and biochemical characteristics of the clones such as rooting depth, vine vigor, ground cover, root neck length, cortex thickness, latex content and concentration of hydroxycinnamic acid esters in the root surface and latex (Stevenson et al. 2009; Muyinza et al. 2012; Anyanga et al. 2013; Osaru et al. 2023). It was recently reported that most sweetpotato morphological features are strongly and negatively linked with the weevil severity, but only storage root neck length effectively reduces weevil damage (Osaru et al. 2023). The significant differences observed in environments could be attributed to differences in rainfall amounts received at the trial sites, soil types, nutrient levels and the prevailing pest and disease situations. According to previous reports, sweetpotato weevils are more harmful under drier conditions since they can reach roots more easily through cracks that emerge when the soil dries out (Smit et al. 2001; Sorensen 2009). Abi-ZARDI is located in an area that is known to receive less rainfall and with higher mean annual temperatures compared to Ngetta-ZARDI (Kaizzi et al. 2012; Sserumaga et al. 2016). This is probably why this trial site recorded higher levels of weevil severity and incidence compared to Ngetta- ZARDI.

Knowledge of heterosis for desirable traits in different crosses is important for the efficient selection of parental genotypes in breeding programs. In the present study, we observed desirable heterosis for weevil severity, and weevil incidence in over 25% of the families whereas significant heterotic gains for storage root yield and dry matter content occurred in over 20% of the families studied. Heterosis occurred in both negative and positive directions for all traits, which implies that both favorable and unfavorable complementary dominant or partially dominant genes were at play (Shrestha et al. 2021). Negative heterosis is usually desirable when it comes to pests and diseases since it indicates that the severity is lower in the progeny compared to the parents or best parent, implying more resistance (Sseruwu 2012). On the other hand, for traits such as storage root yield and dry matter content, positive heterosis is preferable. This study recorded fairly low mid-parent heterotic gains for weevil severity and incidence. This indicates that small increments in resistance to sweetpotato weevils could possibly be achieved through exploiting heterosis in this population. The highest level of MPH observed for weevil severity in this study (− 7.9%) is lower than what was recently reported by Odama (2021), who obtained heterotic gains of up to − 15.5% for weevil severity while studying a diallel population of 202 F_1_ genotypes.

Grüneberg et al. (2015) reported heterotic gains of up to 58.7% in SRY in a sweetpotato population generated from 16 clones, which is much higher than what was estimated in this study. More recently, heterotic gains ranging between 20 and 40% were reported for storage root yield using reciprocal recurrent selection whereas negligible heterosis was observed in other sweetpotato quality traits (Grüneberg et al. 2022). Mid-parent heterotic gains for dry matter content observed in this study were relatively low and ranged from 3.6 to − 6.3%. In comparison to the low levels found in this investigation, Gurmu et al. (2018) found greater amounts of MPH for dry matter content ranging from 3.5 to 48%. In general, higher levels of heterosis indicate a higher likelihood of selecting offspring that perform better than their parents.

The magnitude of heterosis is understood to be a function of the genetic divergence between two parents—in other words, the greater the genetic variability, the more the heterosis displayed (Falconer and Mackay 1996; Grüneberg et al. 2015). It was previously reported that the parental lines used to create the MDP population came from two distinct gene pools, “A” and “B,“ which had been found in the East African sweetpotato germplasm using simple sequence repeat (SSR) markers (David et al. 2018). However, further research on the genetic diversity of these lines using more robust single nucleotide polymorphisms (SNPs) markers and the *I. trifida* reference genome revealed that the gene pool subdivision was not as clear cut as initially presumed, with admixtures observed in the majority of the MDP parental lines (Wu et al. 2018). This probably explains the low levels of heterosis that were observed in this study. According to some earlier studies, the progeny of parents from diverse gene pools displayed high levels of heterosis in sweetpotato (Grüneberg et al. 2009). In order to successfully exploit heterosis in sweetpotato breeding schemes and ensure enhanced genetic gains, there is need for breeders to further develop the existing hybrid populations by including superior clones that are genetically diverse. The continued application of robust genetic markers such as SNPs that clearly differentiate between closely related genotypes is vital in sweetpotato population improvement since they facilitate the selection of diverse parents for controlled crossing in breeding programs. Given that the majority of the parental genotypes employed in this study originated from East Africa, it would probably be worthwhile to investigate or apply heterosis in controlled crosses using parents with different ancestries.

Two parental genotypes that were used in this study and are known to be susceptible to sweetpotato weevil infestation- ‘Mugande’ and ‘Dimbuka-bukulula’, exhibited favorable heterosis and lower means for both weevil severity and incidence. This implies that their progeny displayed more resistance to weevils compared to them. Although being susceptible, these two parental genotypes recently demonstrated higher general combining ability for weevil resistance, and produced offspring with higher levels of resistance to sweetpotato weevils compared to most families (Mugisa et al. 2022). In the present study, we further observed that both mid-parent and high-parent heterotic gains were generally higher among the moderately resistant x susceptible crosses than among the susceptible x susceptible crosses, probably due to resistant parents contributing more to resistance than the susceptible ones. This observation highlights the need for inclusion of the more resistant lines identified through this study as parents in further breeding for resistance to sweetpotato weevils.

One of the main activities in breeding programs is identifying segregants that are superior to their parents for purposes of further testing for potential release as improved varieties or for further deployment as parental genotypes in breeding. In this study, all traits were found to be transgressive. The highest percentage of superior transgressive segregants were obtained for storage root yield (21%) and weevil severity (18%), which suggests that there is a higher possibility of obtaining superior varieties for these two traits compared to the others. According to earlier investigators, the extremely heterozygous character of sweetpotato likely caused either the accumulation or loss and complementary action of favorable alleles from parental lines in their progeny, which could account for the transgressive segregation reported in this work (Falconer and Mackay 1996; Mackay et al. 2021; Tanksley 1993). The levels of transgression segregation shown here might have likely been substantially higher if the parental genotypes had been more diverse. Yada et al. (2017) reported higher levels of transgressive segregation in a population that had a rather high level of genetic variation.

The agronomic qualities of newly released resistant varieties should ideally be at par with or better than those of the susceptible varieties they replace. However, it can be difficult for breeders to advance multiple traits at once. This study revealed that clones expressing resistance to SPWs are generally reporting approximately 10% lower yields compared to their more susceptible counterparts. Expression of resistance against bio-antagonists such as pests and diseases at times comes at a metabolic cost to plants (Brown and Rant 2013). This is due to the fact that it activates inducible defense mechanisms, which redirect some of the plant’s resources and energy away from growth and reproduction and toward self-defense, thus imposing a measurable cost or yield penalty on the host plants (Vos et al. 2013; Vrieling et al. 1991).

There are two major resistance mechanisms that exist in plants: antixenosis (non-preference), the ability of plants to avoid colonization by the pest and antibiosis, the suitability of a plant to the pest (Stenberg and Muola 2017). Both may incur fitness costs and require trade-offs by the plant with other desirable traits. Previous studies on sweetpotatoes have shown that clones may employ both mechanisms in SPW resistance. Some clones exhibit non-preference through features such as deep roots, high vine vigor and heavy pubescence which deter weevils from accessing them (Stathers et al. 2003; Muyinza et al. 2012). Others exhibit antibiosis through the production of hydroxycinnamic acid (HCA) esters (Anyanga et al. 2013; Stevenson et al. 2009). Hydroxycinnamic acids are usually produced by plants for protection against biotic and abiotic stress (Heleno et al. 2015), upon stimulation by the enzyme phenylalanine ammonia lyase (PAL) which is found in plants (Hyun et al. 2011). They are a byproduct of the biochemical pathway that produces lignin, a crucial plant pest defense mechanism (Boerjan et al. 2003; Anyanga et al. 2017). The yield penalty that was observed in this study could probably be attributed to the diversion of metabolic resources towards the enhanced production of HCAs and lignin for plant defense, thereby negatively impacting root growth. Considering that yield is a key trait in sweetpotato improvement, there may have to be a trade-off between maximizing root yields and controlling sweetpotato weevils if a resistant variety is to be successfully bred and released in the region. For instance, sweetpotato breeders might need to prioritize high yields and select for moderate SPW resistance instead of higher resistance if it imposes a yield penalty on the crop. This would enable farmers living in SPW prone areas to profit from both traits in the event that an improved variety is released.

## Conclusion

Weevil severity and incidence, root yield, and dry matter content all showed relatively low levels of heterosis in the MDP population, indicating that the chances of improving sweetpotato through exploitation of heterosis in controlled crosses using parents with generally similar backgrounds is minimal. Transgressive segregation was observed in all traits although crosses ‘Wagabolige’ x NASPOT 10 O and ‘New Kawogo’ x ‘Ejumula’, particularly produced the highest number of superior segregants with regards to weevil resistance. Out of the 64 crosses, only the NASPOT 11 x NASPOT 7 cross demonstrated desirable heterosis and had superior transgressive segregants for all the traits examined. This particular cross is recommended for attention in breeding for weevil resistance through population improvement. The findings of this study further suggest that development of sweetpotato weevil resistance in *Ipomoea batatas* may confer a yield penalty to the crop. There is need for breeders to strike a suitable balance between achieving high yields and weevil resistant clones in order to develop with a variety that is suitable for farmers in weevil-prone areas. The superior clones identified through this study could hereafter be incorporated as parental genotypes for use in population improvement or for establishing superior hybrid breeding populations.

## Data Availability

The data used in this article can be accessed online at: https://sweetpotatobase.org/.
